# Quality of maternal care in the Southeast Asia region

**DOI:** 10.1016/j.lansea.2024.100433

**Published:** 2024-06-19

**Authors:** The Lancet Regional Health – Southeast Asia

Before the onset of the COVID-19 pandemic, there had been marked progress in improving reproductive, maternal, neonatal, and child health (RMNCH) outcomes in the southeast Asia region (SEAR), and most SEAR countries were on track to attain the SDG 3 health targets related to RMNCH outcomes. In this issue of *The Lancet Regional Health—Southeast Asia*, Thomas Gadsden and colleagues show the negative impact of COVID-19 on essential service provision for RMNCH in SEAR. This study documents large disruptions in services across six of 11 countries in SEAR (there were no available data for the remaining five countries). The most disrupted services included antenatal care (ANC), facility-based births, postnatal care, and immunisation services.

ANC services have been a critical area for improving maternal and infant health in the region and significant progress has been made in ensuring coverage of these services; however, this has not fully translated into reduction in mortality outcomes. Studies have long recorded the poor quality of these services as the main factor responsible for poor outcomes in maternal and child survival. A study across 23 countries in various regions of the world demonstrated that institutional delivery and frequent ANC visits had the strongest positive associations with quality postnatal care services for both mothers and neonates.

Dandona and colleagues analysed births in 2020–2021 in the state of Bihar, India, which fares poorest in maternal and child health indicators. The study reports significant inequities in quality of care, with only two in ten ANC visits considered to be of adequate quality. Village Health Nutrition Days, which were designated to provide first contact for primary care, were used by seven in ten women for the first ANC visit; however, women were only checked for blood pressure and weight and therefore there was limited scope for identifying complications or referring women for adequate care. The use of private providers was much higher than the use of public facilities for ANC services in this population despite no difference in quality of care. The authors highlight the need to bolster laboratory services for urine and blood tests to improve ANC services (availability was low in both private and public facilities). Abdomen check-up was significantly more common with the private providers than with the public facilities. This is important as breech position is a significant risk factor for early neonatal death and stillbirth, that could be targeted early if the risk is known. Additionally, calculating gestational age is difficult in areas where routine ultrasonography is unavailable. This impacts small-for-gestational-age and preterm births.

Task shifting from doctors to auxiliary nurse midwifes in public facilities has also been cited as a reason for poor quality of ANC services. In the absence of a midwifery regulatory system, there is a marked gap in training and regulation of the services that midwives are expected to deliver at primary care level. Midwives and nurses become the sole care providers available in the absence of physicians and may be called upon to deliver care interventions without any legal protection. Regulatory mechanisms, infrastructure, training of health workers, retention and motivation of the health work force, and support systems are all important in providing quality care to women. WHO recommends skilled health workers must be trained and retained while strengthening accessible infrastructure that delivers evidence-based, person-centred care.

Facility-based births have been a critical area to reduce neonatal and maternal mortality in the region. We reported in an earlier Editorial how low quality of these services impacted neonatal mortality, leading to deliveries in facilities without the capacity to handle maternal and newborn complications adequately. More women are moving away from institutional care due to disrespect during treatment. In a study on treatment of women during facility-based childbirth, women in India reported limited communication and involvement in their care, with providers failing to explain procedures, medications, and involve them in decision-making. The authors highlight the need for better communication, involvement, and patient-centred care for women during pregnancy and childbirth. Labour induction and augmentation is also not clinically indicated in one third of the cases at public and private facilities in India.

Social norms, cultural contexts, and economic factors play a major role in use of ANC services. For example, stigma associated with adolescent pregnancy prohibits girls and families from seeking care, increasing the risk of mortality in this age group. Health system factors play a major role in driving demand for these services. High-quality governance, management, and community engagement were associated with best performance in providing quality health services.

WHO suggests that quality of ANC is determined by positive pregnancy experience, accessible and affordable services, person-centred treatment, and education and support. Taking quality improvement as a key strategy, WHO SEAR developed the regional framework for improving the quality of care in RMNCH in 2015. The Regional Office introduced the Point of Care Quality Improvement approach in 2016 in collaboration with UN partners and academic institutes. It is time to transform health systems to meet the needs of women, especially during vulnerable periods of life and for those from disadvantaged backgrounds.

The Lancet Group is strengthening its efforts in driving research by advocating Women and health as one of focus areas in 2024. As *The Lancet Regional Health—Southeast Asia* approaches its second anniversary, we are reminded that over 100 women per 100,000 live births still die each year in the region. We invite researchers and programme implementation specialists to join us in shining a light on some of the critical areas that warrant urgent attention in improving quality of ANC and RMNCH services in the region. It is time women get the care they deserve.

